# An Energy‐Corrected Fast Post‐SCF Local‐Hybrid Scheme for Highly Accurate Energy Differences of Large Main‐Group Systems

**DOI:** 10.1002/jcc.70431

**Published:** 2026-06-16

**Authors:** Artur Wodyński, Martin Kaupp

**Affiliations:** ^1^ Institute of Chemistry, Theoretical Chemistry/Quantum Chemistry Technische Universität Berlin Berlin Germany

**Keywords:** DFT, local hybrid functionals, neural‐network local mixing function, strong‐correlation factor, zero‐sum game

## Abstract

Local‐hybrid (LH) density functionals admix exact exchange locally in real space and thereby can be powerful tools to ameliorate the usual zero‐sum game between reducing self‐interaction errors and modeling static correlation. But as with other hybrid functionals the practical use of LHs for large systems is limited by the cost of evaluating exact‐exchange quantities self‐consistently. Here we introduce an energy‐corrected local‐hybrid framework, EC(LH)@(m)GGA, in which a computationally expedient semi‐local reference density is used for a single post‐SCF evaluation with an advanced LH. Using the recent neural‐network‐based LH25nP LH as a prototype, we show that the EC route based on GGA or meta‐GGA orbitals preserves the characteristic accuracy profile of the parent local hybrid and reaches state‐of‐the‐art rung 4 performance on the GMTKN55 test suite (WTMAD‐2 around 2.4–2.7 kcal/mol, depending on grid). The top performance of LH25nP for spin‐restricted bond dissociation as a strong‐correlation measure is retained in this framework. The dominant post‐SCF overhead in timing is governed by the EC grid. For the practical gridsize 3, the total EC(LH)@(m)GGA cost is only about ~2–3× that of a GGA single point, typically about an order of magnitude less than a full LH SCF. Overall, EC(LH)@(m)GGA provides a simple post‐SCF route to state‐of‐the‐art rung‐4 energetics at a cost close to semi‐local DFT, applicable to large systems.

## Graphical abstract

A post‐SCF energy‐corrected local‐hybrid scheme, EC(LH)@(m)GGA, is introduced, in which hyper‐meta‐GGA ingredients obtained from inexpensive meta‐GGA or GGA orbitals are used as input for a single local‐hybrid energy evaluation. Using LH25nP as a prototype, this strategy retains the characteristic accuracy and strong‐correlation improvement of the parent local hybrid, achieving almost unchanged GMTKN55 performance (WTMAD‐2 ≈2.5 kcal/mol). EC(LH)@(m)GGA thus delivers state‐of‐the‐art rung‐4 energetics at near‐semi‐local cost, with an approximately 10× speedup over fully self‐consistent LH calculations.

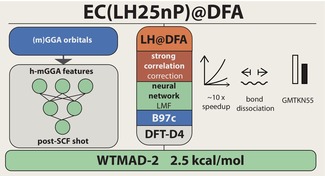

## 1 Introduction

Density‐functional approximations (DFAs) remain the workhorse of electronic‐structure calculations in chemistry and materials science because they combine broad applicability with a favorable accuracy–cost ratio. In particular, semilocal generalized‐gradient approximations (GGAs) deliver robust self‐consistent‐field (SCF) convergence and near‐linear scaling building blocks, making them an attractive default when large systems, extensive conformational sampling, or high‐throughput studies are required.

At the same time, the continuous development of DFAs has highlighted a long‐standing zero‐sum‐game tension: improving one part of chemical space often deteriorates another [1, 2, 3]. For semi‐local DFAs like GGAs or meta‐GGAs, a commonly cited root cause of many failures are self‐interaction errors (SIE) [4]. A standard and broadly successful mitigation strategy is to introduce a fixed (global) fraction of Hartree‐Fock‐like (HF) exchange (“exact exchange,” EXX), leading to global hybrid functionals [5, 6, 7]. This admixture typically reduces SIE and improves nonlocal exchange effects, but it can also worsen the description of strong (static) correlation, which to some extent is mimicked by the semi‐local exchange component [8], and it makes application of such hybrid functionals substantially more expensive, in particular for extended systems. A similar trade‐off pertains to so‐called range‐separated hybrid functionals [9, 10].

Local hybrid (LH) functionals offer a promising route to mitigate the zero‐sum trade‐off by admixing the EXX energy density locally in space, governed by a local mixing function (LMF) [3, 11, 12]. This locality provides a transparent physical lever: the aim is to retain or even enhance the semi‐local DFA exchange in regions where it beneficially models static correlation, while selectively increasing the EXX contribution in other regions to reduce SIE and improve the description of nonlocal exchange effects. Recent constructions of “strong‐correlation‐corrected local hybrids” (scLHs) or their range‐separated extensions (scRSLHs) illustrate this potential. We initially focused on heuristic human‐designed local‐hybrid LMF models, for example, in scLH22t or ωLH25tdE [13, 14, 15, 16, 17]. However, physical constraints on the LMF are known only for the core region and the asymptotics, leaving the chemically crucial valence region comparatively underdetermined. This motivated us recently to treat the LMF as a data‐driven object and to train it using a neural‐network representation [18, 19, 20, 21, 22]. The resulting models, for example LH25nP and LH24n [18, 19], achieve state‐of‐the‐art accuracy for the weak‐correlation‐dominated GMTKN55 benchmark suite [23]. When combined with DFT‐D4 dispersion corrections [24, 25], LH25nP does not only provide the currently lowest WTMAD‐2 value (2.47 kcal/mol) of a rung‐4 functional for GMTKN55, due to its built‐in strong‐correlation factor it also shows improved strong‐correlation signatures such as a correct dissociation limit of spin‐restricted bond dissociation curves. We also note the transfer of the n‐LMF idea to (range‐separated) local double hybrids (DLHs) [20] and most recently to doubly local double hybrids (DLDHs) [22]. In the latter case, LMFs for both exchange and (PT2) correlation are formulated as neural networks.

Calculation of the EXX contribution makes the use of such LHs computationally more expensive compared to simpler semi‐local functionals, even though efficient semi‐numerical integration schemes are nowadays used [26, 27, 28]. The higher computational cost for EXX is a general concern in case of hybrid functionals when aiming at very large systems, in particular towards periodic solids. One direction of research therefore focuses on efforts to use computationally more expedient surrogates for the nonlocality information that EXX admixture provides. A notable recent example is the deep‐learning functional Skala [29], which in its most recent incarnation surpasses the quality of the best global hybrids for weak‐correlation chemistry (e.g., WTMAD‐2 2.80 kcal/mol for GMTKN55) at approximately rung‐3 cost by utilizing some grid‐based nonlocality contributions. Skala almost reaches the performance of the latest LHs or their (currently human‐designed) range‐separated variants (e.g., ωLH25tdE 2.67 kcal/mol [17]), but it currently cannot deal with strong‐correlation situations or the zero‐sum game. Other machine‐learning approaches (e.g., CIDER [30] or models inspired by the holographic electron density theorem [31]) are at an earlier stage of development than the physically motivated spatial interpolation between semilocal exchange and EXX provided by the LH framework.

In this work we want to utilize the excellent energetics provided by LHs like LH25nP while reducing the associated computational burden. The strategy we will follow is to apply the more advanced functional non‐self‐consistently on top of orbitals generated by an inexpensive semi‐local functional. That is, we avoid the recomputation of the most burdensome EXX contributions at each SCF iteration. More specifically, we will look at LH25nP either in its original parametrization or a variant retrained for the use with GGA (PBE [32]) or meta‐GGA (r^2^SCAN) [33] orbitals and density. We refer to this framework as energy‐corrected DFT, more specifically as EC(LH)@(m)GGA where the LH corrects the (m)GGA energy. In particular, we will examine the EC(LH25nP)@PBE and EC(LH25nP)@r^2^SCAN models. To some extent, this is the reverse strategy to the often used HF‐DFT strategy where semi‐local DFT energy calculations are used on top of Hartree‐Fock or hybrid‐DFT orbitals and densities to make use of a compensation between density‐driven and functional‐driven errors, as defined in the density‐corrected DFT (DC‐DFT) framework [34, 35, 36]. We note in passing that while due to the lack of quasi‐exact densities in most practical work HF‐DFT is used as their proxy, recent discussions have pointed to pronounced error compensation between overlocalized HF densities and delocalizing functional errors of the DFA used rather than a proper correction of density‐driven errors only [37, 38, 39, 40, 41] (see Reference [42] for a deviating opinion). Here we focus on a computationally expedient semi‐local functional to generate the orbitals and then apply a much more accurate LH functional to compute the final energy with this density. Obviously, such a strategy will work best when functional‐driven errors are more important than density‐driven ones. This is indeed the case in most areas of application, including most of the wide‐ranging main‐group energetics encompassed by the large and widely used GMTKN55 test suite.

While the computational savings of using hybrid functionals with converged densities from semi‐local functionals are starting to attract attention also in other areas of research [43], a more systematic evaluation for functionals that can come close to chemical accuracy for the target area, such as possible with the highly flexible LH25nP‐D4, does not appear to be available and is the topic of this work. We note in passing that Δ‐machine‐learning approaches to improve upon the energies obtained with standard functionals are yet another possible alternative for achieving cost reductions in the computation of accurate energetics [44, 45, 46, 47].

## 2 Theory

### 2.1 Exchange–Correlation Energy of Local Hybrid Functionals

In an LH, the semi‐local exchange and EXX energy densities are mixed locally by a position‐dependent LMF $a\left(r\right)\in \left[0,1\right]$ [12], or with strong‐correlation corrected LMFs [3, 13, 14, 15, 16, 17, 19] $a\left(r\right)\in \left[-1,1\right]$. A convenient energy expression is

(1) $$E_{\mathrm{xc}}^{\mathrm{LH}}=E_{X}^{\mathrm{ex}}+\int \left[1-a\left(r\right)\right]\left(e_{X}^{\mathrm{sl}}\left(r\right)-e_{X}^{\mathrm{ex}}\left(r\right)+G\left(r\right)\right)\;dr+E_{c}^{\mathrm{sl}}$$

where $E_{X}^{\mathrm{ex}}$ is the EXX energy, $e_{X}^{\mathrm{sl}}\left(r\right)$ is the chosen semi‐local exchange‐energy density, and $E_{c}^{\mathrm{sl}}=\int e_{c}^{\mathrm{sl}}\left(r\right)\;dr$ is the semi‐local correlation energy. $G\left(r\right)$ denotes a gauge correction (calibration function, CF) for the exchange‐energy density. It must satisfy $\int G\left(r\right)\;dr=0$, and is motivated by the fact that a mismatch between semi‐local exchange and EXX energy‐density gauges can otherwise lead to overly repulsive noncovalent interactions [12, 48]. However, we could recently show that a suitably chosen LMF alone, including neural‐network LMFs as in LH24n or LH25nP, can already mitigate this issue even without a CF [18, 19, 49].

The EXX energy is defined as:

(2) $$E_{X}^{\mathrm{ex}}=\int \sum_{\sigma }e_{X,\sigma }^{\mathrm{ex}}\left(r\right)\;dr,$$

with the spin‐resolved EXX energy density in the conventional orbital representation (Coulomb gauge)

(3) $$e_{X,\sigma }^{\mathrm{ex}}\left(r\right)=-\frac{1}{2}\sum_{i,j}^{\mathrm{occ}\left(\sigma \right)}\varphi_{\mathrm{i\sigma}}^{*}\left(r\right)\;\varphi_{\mathrm{j\sigma}}\left(r\right)\int \frac{\varphi_{\mathrm{j\sigma}}^{*}\left(r^{\prime }\right)\;\varphi_{\mathrm{i\sigma}}\left(r^{\prime }\right)}{|r-r^{\prime }|}\;dr^{\prime }.$$

### 2.2 Construction of LH25nP

LH25nP is a strong‐correlation‐corrected LH (scLH) in which the position‐dependent EXX admixture to a PBE exchange‐energy density is governed by a composite LMF [19], built from (i) a fixed, human‐designed strong‐correlation factor $q_{\mathrm{AC}}\left(r\right)$, and (ii) a trained neural‐network LMF (n‐LMF), $a_{n}\left(r\right)$, that controls much of the valence‐space behavior. The sc‐factor has a Padé‐type [15] form that is driven by a real‐space detection variable $z\left(r\right)$, defined as a non‐negative ratio between a semi‐local (modified Becke‐Roussel [50]) exchange‐energy density and the EXX energy density,

(4) $$z\left(r\right)=\max\;\left(\frac{e_{x}^{\mathrm{sl}}\left(r\right)}{e_{x}^{\mathrm{ex}}\left(r\right)}-1,0\right),$$

(5) $$q_{\mathrm{AC}}\left(z\right)=0.5+d\;\frac{{\mathrm{kz}}^{i}}{1+{\mathrm{kz}}^{i}},$$

where d, k, and i are parameters obtained in a prior fit to bond‐dissociation data [15, 19].

The composite nP‐LMF of LH25nP is then defined as

(6) $$a_{\mathrm{nP}}\left(r\right)=1-2\;q_{\mathrm{AC}}\left(r\right)\;\left[1-a_{n}\left(r\right)\right].$$

In strongly correlated regions, $q_{\mathrm{AC}}\left(r\right)>0.5$ reduces the local EXX admixture and can drive the total LMF to negative values, which improves spin‐restricted bond dissociation and other measures of static correlation.

The underlying $a_{n}\left(r\right)$ is represented by a shallow multi‐layer perceptron (three hidden layers with 128 neurons each) with GELU activations and a scaled sigmoid output that maps to $\left[-1,1\right]$ [19]. It uses a hyper‐meta‐GGA feature set evaluated on a molecular integration grid (spin‐resolved densities, gradient invariants, kinetic‐energy densities, and EXX energy densities) and is trained against a composite loss function that includes main‐group thermochemistry and barrier data as well as fractional‐spin (FSE10) constraints to encode static‐correlation signatures. The FSE10 set, introduced in our earlier works [13, 19], contains fractional‐spin configurations of second‐ and third‐period p‐block atoms and is designed to probe static‐correlation errors. As an illustrative example, the carbon atom can be represented by the fractional‐spin configuration

(7) $$\begin{array}{l} \left[\text{core}\right]\;{\left(2p_{x}\alpha \right)}^{0.5-\upsilon }{\left(2p_{y}\alpha \right)}^{0.5-\upsilon }{\left(2p_{z}\alpha \right)}^{0}\\ \;{\left(2p_{x}\beta \right)}^{0.5+\upsilon }{\left(2p_{y}\beta \right)}^{0.5+\upsilon }{\left(2p_{z}\beta \right)}^{0}. \end{array}$$

In the variant used here, υ is varied from −0.5 to 0.0 in steps of 0.1 (0.01 in original LH25nP paper), which connects the fully spin‐polarized and spin‐depolarized limits. For the exact functional, the energy should remain constant along this path, whereas approximate functionals typically show a spurious variation. This deviation provides a direct measure of the fractional‐spin error and is included in the training loss. As discussed previously, incorporating FSE10 into the training helps the LMF reduce such errors, especially in cases where the human‐designed $q_{\mathrm{AC}}\left(r\right)$ indicator does not fully capture intermediate static‐correlation effects.

## 3 Computational Details

Training of the EC(LH25nP)@DFA model where DFA = SVWN [51, 52], PBE [32], and r^2^SCAN [33] was performed with a custom Python workflow based on TensorFlow [53]. All reference orbitals and densities were obtained from self‐consistent DFA calculations in a local development version of Turbomole 7.9 [54, 55, 56]. Unless stated otherwise, def2‐QZVPPD basis sets were employed for the W4‐17 and BH76 data, whereas def2‐QZVP was used for GMTKN55‐type benchmarks [23, 57, 58]. Diffuse functions were added for those GMTKN55 subsets where recommended [23]. Coulomb integrals were accelerated with the RI‐J approximation using Turbomole's “universal” auxiliary basis sets [59, 60, 61].

The EC(LH) energy correction was evaluated *post‐SCF* on top of the converged DFA density using Turbomole 7.9 [54, 55, 56]. Throughout this work, DFA SCF calculations were carried out with a fixed medium‐sized grid (program keyword “gridsize m4”) to ensure a consistent and numerically stable semi‐local density reference. In contrast, the EC(LH) correction was evaluated on reduced numerical grids (sizes 2–4), the size of which directly controls the dominant cost of the EXX‐derived ingredients and enables a systematic accuracy‐cost trade‐off. EXX energy densities entering the feature set and the local‐hybrid correction were obtained by semi‐numerical integration [26, 28, 62], using the semiJK algorithm [27]. All SCF calculations were converged to $10^{-7}$ Hartree.

While $a_{n}\left(r\right)$ (see Theory) was retrained for optimal post‐SCF@DFA performance, the strong‐correlation factor $q_{\mathrm{AC}}\left(r\right)$ and the parameters of the B97 correlation functional were kept fixed at the values adopted in the parent LH25nP construction [19]. The input features were evaluated on molecular integration grids and included standard spin‐resolved semi‐local descriptors together with the EXX‐derived quantities required by the composite LMF. To accelerate convergence and preserve continuity with the parent functional, the EC(LH25nP) optimization was initialized from the published LH25nP network parameters; specifically, the LH25nP weights and biases were used as starting values and then fine‐tuned within the EC(LH)@DFA protocol [19].

The training procedure was carried out simultaneously on three integration grids (size 2–4). The training set consisted of FSE10, W4‐17 atomization energies, BH76 barrier heights, and dietGMTKN55, with weights of 0.2, 0.5, 1.0, and 1.0, respectively [13, 19, 63, 64, 65].

Validation and model selection were based on Slim20‐GMTKN55 [66]. This subset was used exclusively for validation and for selecting the final model, whereas the details of the selection protocol are beyond the scope of the present work. Final performance was assessed on the full GMTKN55 benchmark suite [23]. We note in passing that in Reference [19] possible overtraining of the n‐LMF in LH25nP has been evaluated by eliminating training data from the GMTKN55 evaluation and was found to be small. It is expected that fine‐tuning for a given density will not change this.

Spin‐restricted covalent bond‐dissociation curves for the DISS10 set [14] were evaluated using gridsize 4 with diffuse 2 and def2‐QZVPPD basis sets in order to maintain consistency with previous work [13, 14, 15, 16, 17, 19].

All LH energy calculations on sets like GMTKN55 or Slim20‐GMTKN55 were done with atom‐additive DFT‐D4 dispersion corrections [24, 25], using the published D4 parameters of LH25nP‐D4 [19] (see below).

Timing comparisons for oligoacenes used def2‐QZVP basis sets, those on ubiquitine def2‐TZVP basis sets.

## 4 Results

### 4.1 Density Sensitivity Evaluation of LH25nP‐D4@DFA Energies for GMTKN55

To estimate the density‐sensitivity of LH25nP energies [67], we compare WTMAD‐2 values for the full GMTKN55 suite and its subcategories obtained from fully self‐consistent LH25nP calculations with added D4 corrections (LH25nP‐D4@LH25nP) with values obtained by evaluating LH25nP‐D4 non‐self‐consistently on SVWN, PBE, or r^2^SCAN orbitals and densities (LH25nP‐D4@DFA). The results are summarized in Table 1, including self‐consistent literature values for the three (semi‐)local DFAs. Statistics for the individual 55 subsets are provided in Tables S1–S3. We note in passing that often density sensitivity is defined as the energy difference between computations using the LDA and HF densities [41]. This would give larger overall differences but is expected to follow the same trends as the numbers analyzed here.

**TABLE 1:** WTMAD‐2 for GMTKN55 and its subcategories in kcal/mol for LH25nP evaluated self‐consistently (LH25nP‐D4@LH25nP), non‐self‐consistently on different DFA densities (LH25nP‐D4@DFA where DFA = SVWN, PBE and r^2^SCAN), and corrected by the EC(LH25nP‐D4)@DFA procedure at different grid sizes (g2, g3, g4).

	Basic & small	Iso & large	Barriers	Intermol. NCIs	Intramol. NCIs	GMTKN55
LH25nP‐D4^a^	2.11	2.93	2.44	2.27	2.94	2.48
SVWN‐D3(BJ)^b^	36.00	27.70	47.30	30.10	38.00	34.00
LH25nP‐D4@SVWN^c^	2.49	4.13	3.34	6.22	3.53	3.82
EC(LH25nP)‐D4@SVWN^c^						
g2	2.22	3.10	2.42	2.79	3.79	2.81
g3	2.16	2.98	2.50	2.68	2.86	2.58
g4	2.13	2.96	2.30	2.72	2.56	2.49
PBE‐D3(BJ)^b^	8.70	5.00	10.10	8.10	10.40	7.70
LH25nP‐D4@PBE^c^	2.27	3.32	3.06	3.75	3.35	3.05
EC(LH25nP)‐D4@PBE^c^						
g2	2.23	2.95	2.22	2.49	3.70	2.68
g3	2.09	2.98	2.44	2.45	3.00	2.53
g4	2.11	2.83	2.37	2.24	2.66	2.39
r^2^SCAN‐D4^d^	7.54	5.55	8.26	14.29	7.46	5.74
LH25nP‐D4@r^2^SCAN^c^	2.16	2.92	2.83	2.85	3.17	2.70
EC(LH25nP)‐D4@r^2^SCAN^c^						
g2	2.20	2.95	2.21	2.35	3.61	2.62
g3	2.03	2.77	2.40	2.17	3.09	2.43
g4	2.08	2.85	2.32	2.10	2.74	2.37

*Note:* All LH25nP‐based results have been obtained with the same DFT‐D4 parameters (see text). For comparison, literature values for the (semi‐)local DFAs are also shown.

^a^ Reference [19].

^b^ Reference [23].

^c^ This work.

^d^ Reference [68].

Starting with the overall WTMAD‐2 values for the full suite, we see that use of the SVWN, PBE, and r^2^SCAN density causes an increase by 1.34, 0.57, and 0.22 kcal/mol, respectively (Table 1). That is, the additional energy errors caused by these more approximate densities decrease from the LDA to the GGA and meta‐GGA densities, following expectations of the usual Jacob's ladder hierarchy [69]. Clearly, the error introduced by evaluating LH25nP‐D4 on the approximate PBE density is moderate even without any reparametrization for the EC protocol, and that for the r^2^SCAN density is even smaller. Given the much higher deviations of the (semi‐)local DFAs themselves, the results confirm the small density sensitivity of the GMTKN55 energetics overall, consistent with the reported dominance of functional‐driven over density‐driven errors [34, 35, 36]. Notably, the overall WTMAD‐2 values of LH25nP‐D4@PBE and LH25nP‐D4@r^2^SCAN are lower than those of most known rung‐4 functionals [19], in the latter case also below the most recent update for the deep‐neural‐network functional Skala [29].

In general, the density sensitivity is by far largest for the intermolecular non‐covalent interactions (NCIs), but even in this case it is already moderate for LH25nP‐D4@r^2^SCAN. For the LDA density, the second‐largest density sensitivity is seen for the iso & large subcategory followed by barriers and intramolecular NCIs, with smaller effects for basic & small. With PBE orbitals the second‐largest effect is for barriers, but the changes are already reduced significantly. Finally, with r^2^SCAN orbitals, the changes relative to the self‐consistent results are already minor for most subcategories and above 0.5 kcal/mol only for the abovementioned intramolecular NCIs.

Closer analyses of the impact of density sensitivity of different subsets on overall GMTKN55 performance would have to include the different weighting factors for the subsets, and we refrain from detailing this. However, looking at the largest changes with density for individual reactions, we see that those occur within the WATER27 and MB16‐43 subsets. The former is part of the intermolecular NCIs that also are the overall most affected subcategory. Hydrogen bonding in water, from clusters to the liquid, has been discussed as a case where HF‐DFT is very successful [41, 70, 71]. For the “mindless benchmarking” MB16‐43 subset [23], unusual and difficult bonding situations may be encountered, which may explain the observed, somewhat larger density sensitivity for some reactions in this subset.

We chose to use the D4 parameters of LH25nP without reoptimization. The reason is, that with a flexible functional like LH25nP, training of the LMF in the presence of the D4 terms is expected to absorb any small effects that might occur due to the D4 coefficients not being optimal. Such a philosophy is also followed with deep‐neural network functionals like Skala or DM21 [29, 72]. To test this assumption, we have evaluated the effect of reoptimizing the D4 parameters for the two cases LH25nP‐D4@SVWN and LH25nP‐D4@PBE. While the overall WTMAD‐2 value for the LDA‐based calculations was reduced slightly by 0.37 kcal/mol, with the PBE density it even increased by 0.09 kcal/mol. We attribute these effects to a subtle interplay between functional and dispersion term, which in the latter case reveals some error compensation. For all practical purposes, these data suggest that reoptimization of dispersion corrections is not required in the context of the EC(LH) scheme, as we will not recommend use of SVWN densities.

### 4.2 EC(LH25nP) LMF Retraining and Evaluation

While LH25nP‐D4@PBE and in particular LH25nP‐D4@r^2^SCAN do already provide almost top rung‐4 accuracy for GMTKN55, we now proceed to evaluate to what extent a fine‐tuning of the n‐LMF in LH25nP can be used to provide even more accurate energies. For the EC(LH25nP‐D4)@DFA scheme we retrained the LMF for the given density with input features obtained jointly for gridsize 2, 3, and 4. In this way it is hoped that some degree of flexibility with respect to the grid used in the final EC(LH) energy evaluation is embedded within the LMF. After retraining, evaluation of EC(LH25nP)‐D4@DFA is done individually for each of these three grid sizes for the full GMTKN55 test suite (we also considered gridsize 1 but found larger deterioration). The underlying SVWN, PBE, or r^2^SCAN densities are in all cases computed accurately using gridsize m4.

The post‐DFA evaluation of the EXX energy density, $e_{X}^{\mathrm{ex}}\left(r\right)$, enters both the LMF and the LH in general. In a straightforward grid‐based semi‐numerical implementation, building $e_{X}^{\mathrm{ex}}\left(r\right)$ scales as $O\;\left(N_{\mathrm{AO}}^{2}\cdot N_{\text{grid}}\right),$ as for each grid point one effectively contracts products (or equivalent intermediates) over pairs of atomic orbitals (AOs). Since $N_{\text{grid}}$ grows linearly with the chosen numerical integration grid, reducing the grid size is a straightforward lever to lower the prefactor of the EC correction, which is particularly relevant for large systems and extended basis sets, where $N_{\mathrm{AO}}$ is substantial. Therefore we will explore the effect of grid size on the quality of the EC(LH) correction.

The final WTMAD‐2 evaluation results of the EC(LH25nP‐D4)@DFA scheme with different grid sizes for the full GMTKN55 suite and its subcategories are included in Table 1. Starting with the full set, we see that (a) in all cases the retraining lowers the overall WTMAD‐2 value, which gets close to the self‐consistent LH25nP‐D4 result, and (b) larger grids lower the values further. Indeed, with gridsize 4, the EC(LH25nP‐D4)@PBE and EC(LH25nP‐D4)@r^2^SCAN values are slightly lower than the self‐consistent one, while all values remain above the latter with gridsize 2, with the expected order LDA > GGA > meta‐GGA. The intermediate gridsize 3 appears to be a good choice in all cases to return almost exactly to the self‐consistent WTMAD‐2 value. At this EC(LH25nP‐D4)@DFA level, even the values for the subcategories tend to deviate almost negligibly from their self‐consistent counterparts, except the intermolecular NCIs based on the SVWN density and the intramolecular NCIs based on the PBE density. In view of the above observations for the uncorrected LH25nP‐D4@DFA performances, the largest improvements by the energy‐correction scheme are obviously seen for the intermolecular NCIs (Table 1). That is, the fine‐tuning of the n‐LMF of LH25nP to the given underlying density is clearly able to compensate for most deviations due to density sensitivity within GMTKN55.

During the early retraining experiments we found that inclusion of larger grids than gridsize 4 during training did not improve subsequent evaluation results for the Slim20‐GMTKN55 set with smaller grids. Given the aim of this work to provide a computationally inexpensive scheme, more detailed screening of larger grids was not attempted here.

Closer examination of the results for individual GMTKN55 subsets indicates that the EC(LH) corrections tend to move the MADs closer to those of the self‐consistent LH25nP‐D4 data (Tables S1–S3). But the extent of this improvement depends somewhat on the subset and on the grid. The overall grid‐size dependence is low, however. Only a few subsets exhibit somewhat larger deviations from the self‐consistent results, while some subsets actually obtain smaller deviations than the self‐consistent calculations, suggesting some error cancelation to be operative. For noncovalent interactions, the EC correction shows a clear and largely consistent trend toward improving over the LH25nP@DFA baseline, with the detailed grid dependence reflecting the sensitivity of dispersion‐ and exchange‐dominated descriptors to numerical resolution typically landing close to or better than the self‐consistent LH25nP values. A particularly instructive case is IDISP with PBE or r^2^SCAN densities (Tables S1–S3): the coarse EC gridsize 2 deteriorates the MAD, whereas gridsize 3 or 4 essentially recovers the self‐consistent behavior, consistent with the notion that intramolecular dispersion and conformational energetics are especially sensitive to the faithful evaluation of EXX‐derived local descriptors in low‐density regions, where the grid is sparse. Overall, these patterns support the practical conclusion drawn above: EC gridsize 3–4 generally offer the most robust behavior for challenging subsets while retaining a substantially reduced overhead compared to a fully self‐consistent local‐hybrid treatment (see below). We note that C60ISO exhibits an exceptionally large grid‐size dependency (Tables S1–S3) with all input densities. However, this has nothing to do with the EC(LH) retraining but is found already for self‐consistent calculations with LH25nP‐D4 (MAD 21.3, 10.9, 15.0 kcal/mol for gridsize 2, 3, 4, respectively). Closer analysis shows that this high grid dependence arises from the human‐designed strong‐correlation factor included in the LMF. Indeed, we recently found as part of a re‐evaluation of the C60ISO reference data [73] that strong‐correlation terms in LHs and their range‐separated variants show appreciable real‐space contributions outside the C_60_ cage that affect the relative energies of C60ISO.

Figure S1 compares, for all GMTKN55 reactions, the reference‐data errors of non‐self‐consistent LH25nP‐D4@DFA and EC(LH25nP‐D4)@DFA calculations with the corresponding self‐consistent LH25nP‐D4 errors, for DFA = PBE and r^2^SCAN. Points close to the diagonal indicate agreement with the self‐consistent results, whereas points closer to the horizontal axis indicate smaller errors relative to the reference data. The interesting aspect here is, that for those points with the largest deviations from the reference data, the LMF fine‐tuning tends to give even smaller deviations from reference data than the self‐consistent LH25nP‐D4 calculations. This may reflect the fact that in the EC(LH)@DFA scheme we use more directly the same type of features in training and evaluation, without the intermediate step of an SCF procedure.

A separate measure of performance for a large set of main‐group reaction energies is the W4‐11RE set of ca. 11,000 reactions generated from the 140 atomization energies of the W4‐11 set [74]. Notably, while W4‐11 is part of the training set, rankings of different functionals for the W4‐11RE energies are often found not to match those for W4‐11. LH25nP‐D4 provides currently the clearly lowest MAD for W4‐11RE of 2.4 kcal/mol [19]. Table S4 shows that LH25nP‐D4@DFA with SVWN, PBE, or r^2^SCAN densities gives MADs that are only 0.3–0.5 kcal/mol higher. Fine‐tuning of the n‐LMF in the EC scheme provides 2.2 kcal/mol, that is, a value that is even slightly below the self‐consistent one.

Figure S2 illustrates the shapes of the original and retrained LMFs for CS, N_2_, and for the prototypical NCI cases Ar_2_ at a separation of 7.5 bohr and (CH_4_)_2_ at 7.0 bohr, where gauge‐related effects usually become particularly apparent. For CS and N_2_, the changes relative to the original LH25nP form remain small in the valence and bonding regions. Somewhat surprisingly, however, the retrained LMFs tend to adopt slightly smaller values there. At first sight this appears counterintuitive, since one might expect that the larger density error associated with orbitals from (semi‐)local functionals would favor an increased contribution from EXX in the LMF. We suspect that this behavior reflects a subtler fine‐tuning effect whose detailed origin requires further study. By contrast, larger changes are observed in the asymptotic region, most pronouncedly for EC(LH25nP)@SVWN. This is noteworthy because the asymptotic part of the LMF is expected to contribute to the marked improvement in the NCI category when going from LH25nP‐D4@DFA to EC(LH25nP‐D4)@DFA. It therefore appears plausible that the retraining improves the balance between density‐driven and energy‐driven errors. Indeed, for the two NCI examples, a noticeable lowering of the LMF is also found in the region between the two monomers (Figure S2), together with a less flat profile than in the original LH25nP. This is again most pronounced for EC(LH25nP)@SVWN. At the same time, the LMF in this region still remains sizeable, in clear contrast to the near‐zero values found there for the t‐LMF in LH20t. In that case, the quenching of the LMF in the intermolecular region was previously identified as an important contributor to the overly repulsive potential‐energy surface for this system and to the associated gauge problem [18, 49]. One may speculate that the present behavior is partly related to error compensation across different grids, since the retrained models were fitted jointly on gridsize 2–4, including coarser grids that provide a less balanced description of NCI regions, whereas the original version was effectively optimized for NCI behavior only on gridsize 4. At present, however, it seems more likely that this behavior reflects the fine‐tuning required to balance density and energy errors simultaneously.

### 4.3 Performance of LH25nP@DFA and EC(LH25nP)@DFA for Strong‐Correlation Situations

Most functionals such as global or range‐separated hybrids provide a far too high asymptotic energy in the spin‐restricted dissociation of covalent bonds [3]. The energy difference from the sum of the unrestricted separated atoms is twice the so‐called fractional‐spin error (FSE) of these atoms [8], which is a measure of strong correlations. This is part of the zero‐sum game between reducing delocalization and strong‐correlation errors, mentioned in the introduction [1, 2, 3]. LHs and RSLHs with strong‐correlation factors inside their LMFs are among the most promising ways out of this dilemma, and due to its strong‐correlation factor LH25nP belongs into this class of functionals [19, 21]. We note in passing, that semi‐local functionals like the PBE GGA have somewhat reduced FSEs compared to many hybrid functionals but in turn exhibit large delocalization errors. We have seen above that EC(LH25nP)@DFA can retain the excellent overall energetics of LH25nP for situations with predominantly weak correlations. To see if this extends also to strong‐correlation cases like spin‐restricted bond dissociation, we have evaluated the scheme also for such situations. This is done by computing (a) the asymptotic large‐distance limits of such dissociation curves for the 10 main‐group diatomics of the DISS10 set [14], and additionally (b) the height of the unphysical local maxima (“humps”) of these 10 curves relative to the asymptotic limit (HUMP10 set). The latter data set is related to unphysical behavior in the intermediate‐strength correlations at these intermediate distances in the spin‐restricted dissociation curves [14]. Table 2 compares the results for LH25nP@DFA and EC(LH25nP)@DFA calculations to self‐consistent LH25nP and DFA data, while Figure S3 provides the complete potential‐energy curves for the ten diatomics of DISS10.

**TABLE 2:** Mean absolute errors (MAE) and mean signed errors (MSE) for the DISS10 and HUMP10 strong‐correlation sets in kcal/mol using various approaches.^a^

Method	Subset	MAE	MSE
LH25nP^b^	DISS10	18.4	3.9
	HUMP10	27.1	—
LH25nP@SVWN	DISS10	18.4	18.4
	HUMP10	19.7	—
EC(LH25nP)@SVWN	DISS10	7.4	2.8
	HUMP10	28.3	—
SVWN	DISS10	51.9	51.9
	HUMP10	–^c^	–^c^
LH25nP@PBE	DISS10	20.4	20.4
	HUMP10	19.9	—
EC(LH25nP)@PBE	DISS10	8.5	3.5
	HUMP10	29.2	—
PBE	DISS10	61.2	61.2
	HUMP10	–^c^	–^c^
LH25nP@r^2^SCAN	DISS10	20.8	20.8
	HUMP10	20.3	—
EC(LH25nP)@r^2^SCAN	DISS10	7.4	4.6
	HUMP10	30.2	—
r^2^SCAN	DISS10	86.7	86.7
	HUMP10	–^c^	–^c^

^a^ For both SCF(DFA) and post‐SCF EC(LH25nP)@DFA calculations, gridsize 4 with an additional diffuse 2 setting was used to be consistent with previous LH25nP calculations [19].

^b^ Reference [19].

^c^ No local maximum is present.

Starting with the statistical data in Table 2, we see that uncorrected LH25nP@DFA tends to reproduce the self‐consistent LH25nP DISS10 MAE but gives a positive MSE of the same magnitude. This is due to the fact that after the unphysical local maximum, the uncorrected curves in several systems do not come down to the dissociation limit of LH25nP@LH25nP (Figure S3). That is, some part of the much larger FSE that the curves for all three semi‐local functionals exhibit, seems to be density‐driven and is not removed completely, except for the B_2_ and P_2_ dimers, where the LH25nP@DFA curves are close to the reference curve. In these two cases, the EC scheme changes very little. In the other eight cases, the curves are shifted down by LMF‐retraining, either close to the reference curve (O_2_, P_2_, Cl except for the SVWN density), or overshooting and thereby giving an even smaller fractional‐spin error than the reference curve (Al_2_, F_2_, Cl_2_ with SVWN orbitals). As a result, the final DISS10 MAE of EC(LH25nP)@DFA is in all cases smaller than that of LH25nP@LH25nP. We note in passing that the SVWN curve is generally more negative than all other curves, likely signaling overbinding. Here LH25nP@SVWN and EC(LH25nP)@SVWN shift the curve up and make it more realistic around equilibrium but seemingly too high in the asymptote. Overall, the EC(LH25nP)@DFA results are relatively close to the self‐consistent LH25nP data while improving fundamentally over the curves of the underlying (semi‐)local functionals. In most cases, functional‐driven errors seem to dominate here as well, as could have been suspected from Becke's earlier post‐SCF observations with the strong‐correlation‐corrected B13 functional [75]. It is instructive to compare the DISS10 data to those obtained with standard (global, range‐separated or local) hybrid functionals. Just to give an example, the “uncorrected” LH24n LH gives a DISS10 MAE about 15× larger than with LH25nP. Here PBE gives, for example, “only” a ca. 7× larger MAE compared to LH25nP, reflecting detrimental effects of EXX in this context, unless properly modulated locally by real‐space strong‐correlation factors.

### 4.4 The NaCl Dissociation Curve, a Density‐Sensitive Case

While the approaches of this work are clearly intended to be used only for “normal” systems, where functional‐driven errors dominate over density‐driven ones, in this section we provide some results for a prototypical “density‐sensitive case,” the dissociation curve of the ionic NaCl molecule, or more precisely the energy difference between stretched NaCl at a bond length of 6.4 Å and the structure near equilibrium at 2.4 Å. Similar quantities have been analyzed frequently in the context of HF‐DFT [76, 77, 78]. Figure S4 shows deviations from accurate CCSD(T) reference data [78] for the self‐consistently computed DFAs SVWN, PBE, r^2^SCAN, LC‐ωPBE, and LH25nP, as well as HF, for LH25nP@DFA with densities obtained at these levels, and after LMF‐fine‐tuning in the EC(LH25nP)@DFA scheme with the (semi‐)local DFAs.

As expected, the (semi‐)local functionals produce large deviations, interestingly PBE even more so than SVWN. LH25nP@DFA energies provide generally a significant improvement. LH25nP@PBE gives the largest improvement and performs better than HF. Interestingly, even LH25nP@HF improves over self‐consistent HF energies and gives the overall lowest deviations. LC‐ωPBE overlocalizes, LH25nP@LC‐ωPBE corrects in the opposite direction. In all cases LH25nP@DFA is a reasonable approach towards this energy difference, in spite of the assumed large density‐driven errors of the underlying (semi‐)local functionals. In contrast to the GMTKN55 results, for this particular example EC(LH25nP)@DFA is not an improvement over LH25nP@DFA but increases deviations, for all of the densities from (semi‐)local functionals.

### 4.5 Computational Cost of EC(LH)@DFA

**FIGURE 1:**
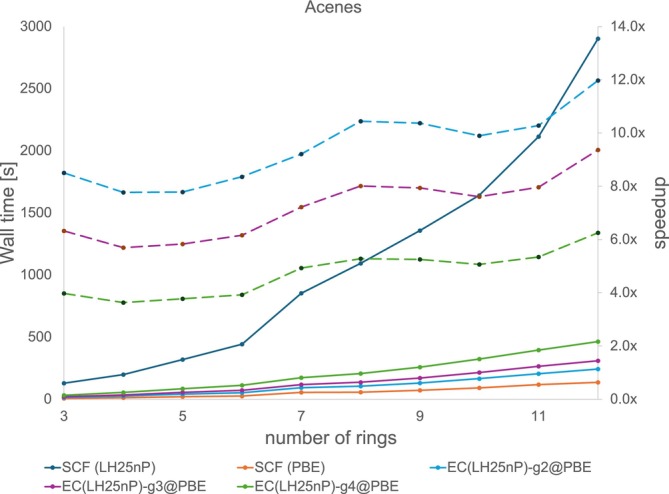
Wall times in seconds (solid lines) and speedup factors relative to self‐consistent LH25nP calculations (dashed lines) obtained with different approaches for oligoacenes as a function of the number of rings.

Figure 1 summarizes the wall times and corresponding speedups compared to self‐consistent LH25nP calculations for a variety of approaches for a series of oligoacenes containing 3–12 rings in $D_{2h}$ symmetry. Table S5 summarizes the underlying data used to construct Figure 1, as well as data for the C_60_ molecule without symmetry. We focus here on the EC(LH25nP)@PBE case but note that the timings of the EC procedure will be the same for, for example, use of r^2^SCAN orbitals. As the self‐consistent r^2^SCAN computation tends to cost at most about 10%–30% more than with PBE for larger systems, consistent with the additional evaluation of the kinetic‐energy density τ (see also Table S5), this extra cost would have to be added to the overall cost of the procedure in the EC(LH25nP)@r^2^SCAN case. All calculations were carried out on 10 cores of an Intel Xeon E5‐2630 v4 CPU, and the reported values correspond to the wall time required for a full single‐point energy calculation. To provide a consistent baseline, Table S5 reports timings for the self‐consistent PBE, r^2^SCAN, and LH25nP calculations together with the corresponding number of SCF cycles required for convergence in each case. Deviations from a monotonous timing increase and smooth speedup curves around 6–8 rings can be attributed to the increased number of SCF iterations required for convergence in the larger systems.

The cost of the EC(LH) correction increases approximately linearly with grid size. For gridsize 2, the correction is slightly cheaper than a self‐consistent GGA calculation, yielding an overall speedup by about a factor of 8–13 relative to the full LH25nP SCF treatment. For gridsize 3, which appears to be a robust choice (see above), the speedup remains about a factor of 6–10 (closer to 10 for the largest systems). The total cost of the EC(LH) procedure is therefore only somewhat more than twice that of a self‐consistent GGA calculation. In all cases, it still provides roughly an order‐of‐magnitude reduction in computational cost compared to a fully self‐consistent LH25nP calculation (Table S5), while preserving its state‐of‐the‐art performance for energetics. Scaling with system size is also reduced.

To assess the possible practical usefulness of this approach, we also report timings for a small protein. As a representative example, we considered ubiquitin with 58 water molecules, containing 1405 atoms. The calculations were carried out with the COSMO solvent model and def2‐TZVP basis sets, that is, using 40,537 basis functions. For the PBE SCF step, gridsize 3 was used, as well as 15 SCF iterations. All timings were obtained on 10 cores of an Intel Xeon E5‐2630 v4 CPU. The PBE SCF part required 22 h, whereas the EC(LH25nP) correction took 13, 23, and 43 h for gridsize 2, 3, and 4, respectively. Thus, a single‐point energy for such a protein with an accuracy approaching that of double hybrids can be estimated within about 1.5 days using only modest computational resources. We did not perform a fully self‐consistent LH25nP calculation for this system, but based on the previous discussion its cost may be estimated to be on the order of 2 weeks.

## 5 Summary and Conclusions

Local hybrid functionals provide currently the top performance for main‐group energetics among rung‐4 functionals as exemplified by the GMTKN55 benchmark suite. When combined with DFT‐D4 dispersion corrections, the recent LH25nP based on a combined neural‐network local mixing function and a human‐designed strong‐correlation factor gives a WTMAD‐2 value for GMTKN55 of 2.47 kcal/mol. At the same time, LH25nP has improved performance for strong‐correlation situations, as exemplified by spin‐restricted dissociation curves for covalent bonds. It therefore belongs to a promising class of recent functionals that escape to some extent the usual zero‐sum game between trying to minimize delocalization and strong‐correlation errors. Similar to other rung‐4 functionals, local hybrids require the computation of exact‐exchange integrals, which makes their use for larger systems more computationally demanding than calculations with semi‐local functionals.

Here we have demonstrated a non‐self‐consistent scheme that preserves the outstanding energetics of LH25nP‐D4 while reducing computing times for energy calculations by an order of magnitude. The proposed scheme of “energy‐corrected DFT” uses orbitals and density from a computationally less demanding semi‐local functional like PBE or r^2^SCAN and then computes the energy based on this density using the local hybrid. Even without any corrections, LH25nP‐D4@PBE(LH25nP‐D4@r^2^SCAN) provide WTMAD‐2 values for GMTKN55 that are only about 0.57(0.27) kcal/mol above the self‐consistent LH25nP‐D4 result. The largest losses in accuracy for these levels, 1.52(0.58) kcal/mol, are seen for intermolecular noncovalent interactions. To correct for this loss in accuracy, we have retrained the neural‐network local mixing function that largely determines the position‐dependent exact‐exchange admixture in LH25nP for this post‐DFA use. While retraining is performed simultaneously for different integration grid sizes, evaluation is then performed separately for different grids.

The obtained WTMAD‐2 values are generally very close to the 2.5 kcal/mol accuracy of self‐consistent LH25nP‐D4 calculations, with overall only a moderate grid‐size dependence. The cost of these energy calculations is only about twice that of a self‐consistent PBE calculation, easily an order of magnitude less than a self‐consistent local‐hybrid calculation. Such an EC(LH25nP)@DFA scheme offers therefore state‐of‐the‐art rung‐4 main‐group energetics at reduced cost, making the routine application to large systems feasible. In addition, EC(LH25nP)@DFA preserves the “beyond‐zero‐sum” performance of LH25nP in the proper spin‐restricted dissociation of covalent bonds. The transferability of LH25nP to transition‐metal systems is still slightly behind that of some other local hybrid functionals, but work towards improved functionals, for example, by improved neural‐network local mixing functions should provide wider accuracy also in that area.

The energy‐corrected DFT scheme suggested here uses the inverse of the idea of the popular HF‐DFT scheme. In the latter one uses orbitals from Hartree‐Fock or functionals with small delocalization errors and then applies a cheaper semi‐local functional for the energy, seeking to either correct density‐driven errors or, as it seems, often benefit from cancelation between density‐driven and functional‐driven errors. Here we use instead a cheaper functional for the orbitals and then improve the energies post‐SCF using a more sophisticated one. Obviously, this scheme is most promising for cases where density‐driven errors are small and we correct mostly functional‐driven errors, which is the case for the vast majority of molecules and possibly solids as well. Yet we have shown that even in a case with large density sensitivity such as stretched NaCl, LH25nP@DFA can provide large improvements over the performance of the underlying (semi‐)local DFAs. This suggests that in such cases we can benefit from error compensation, but in the opposite way of HF‐DFT. Similarly to HF‐DFT, the energy‐corrected DFT scheme would require additional implementation work to access analytic gradients and other properties beyond energies and energy differences. This has not been the aim of this work which has focused on single‐point energies.

## Funding

This study was supported by Deutsche Forschungsgemeinschaft (KA1187/14‐2).

## Conflicts of Interest

The authors declare no conflicts of interest.

## Supporting information


**Table S1:** Mean absolute deviations (kcal/mol) for GMTKN55 subsets with self‐consistent LH25nP (LH25nP@LH25nP; gridsize m4), LH25nP (gridsize 4) on SVWN orbitals (LH25nP@SVWN), and the energy‐corrected LH25nP variant on PBE orbitals (EC(LH25nP)@SVWN), using gridsize 2, 3, and 4. All values include D4 dispersion corrections.
**Table S2:** Mean absolute deviations (kcal/mol) for GMTKN55 subsets self‐consistent LH25nP (LH25nP@LH25nP; gridsize m4), LH25nP (gridsize 4) on PBE orbitals (LH25nP@PBE), and the energy‐corrected LH25nP variant on PBE orbitals (EC(LH25nP)@PBE), using gridsize 2, 3, and 4. All values include D4 dispersion corrections.
**Table S3:** Mean absolute deviations (kcal/mol) for GMTKN55 subsets with self‐consistent LH25nP (LH25nP@LH25nP; gridsize m4), LH25nP (gridsize 4) on r^2^SCAN orbitals (LH25nP@r^2^SCAN), and the energy‐corrected LH25nP variant on PBE orbitals (EC(LH25nP)@r^2^SCAN), using gridsize 2, 3, and 4. All values include D4 dispersion corrections.
**Figure S1:** Correlation between errors relative to the reference values for all reactions in GMTKN55.
**Table S4:** Performance of selected rung 4 functionals in self‐consistent or post‐SCF calculations for the mean absolute deviation (MAD) of the W4‐11RE reaction‐energy test set.
**Figure S2:** Comparison of nP‐LMF plots obtained with the EC(LH) protocol using PBE orbitals and from fully self‐consistent LH25nP calculations.
**Figure S3:** Spin‐restricted potential energy curves for selected diatomic molecules.
**Figure S4:** Errors in the NaCl stretching energy, ΔE_error_[NaCl(2.4˚A) − NaCl(6.4˚A)], in kcal mol^−1^, evaluated with respect to CCSD(T) reference data.
**Table S5:** Wall times (s), number of SCF cycles, and speed‐up of EC(LH25nP)@DFA compared to self‐consistent LH25nP for energy calculations on a series of oligoacene systems with increasing number of rings (in *D*_2h_ symmetry) and for C_60_ in *C*_1_ symmetry.

## Data Availability

The data that support the findings of this study are available in the Supporting Information of this article. The neural network training code nLMFs is publicly available at https://github.com/awodynski/nLMFs_batches. The weights and biases of the retrained n‐LMF are available at https://doi.org/10.5281/zenodo.19554658.
