# Supplementary material for: An Energy‐Corrected Fast Post‐SCF Local‐Hybrid Scheme for Highly Accurate Energy Differences of Large Main‐Group Systems
Source: J Comput Chem. 2026 Jun 16;47(17):e70431. doi: 10.1002/jcc.70431 (PMC13270780; doi:10.1002/jcc.70431)
Supplement: Supplementary file 1 — Table S1: Mean absolute deviations (kcal/mol) for GMTKN55 subsets with self‐consistent LH25nP (LH25nP@LH25nP; gridsize m4), LH25nP (gridsize 4) on SVWN orbitals (LH25nP@SVWN), and the energy‐corrected LH25nP variant on PBE orbitals (EC(LH25nP)@SVWN), using gridsize 2, 3, and 4. All values include D4 dispersion corrections. Table S2: Mean absolute deviations (kcal/mol) for GMTKN55 subsets self‐consistent LH25nP (LH25nP@LH25nP; gridsize m4), LH25nP (gridsize 4) on PBE orbitals (LH25nP@PBE), and the energy‐corrected LH25nP variant on PBE orbitals (EC(LH25nP)@PBE), using gridsize 2, 3, and 4. All values include D4 dispersion corrections. Table S3: Mean absolute deviations (kcal/mol) for GMTKN55 subsets with self‐consistent LH25nP (LH25nP@LH25nP; gridsize m4), LH25nP (gridsize 4) on r2SCAN orbitals (LH25nP@r2SCAN), and the energy‐corrected LH25nP variant on PBE orbitals (EC(LH25nP)@r2SCAN), using gridsize 2, 3, and 4. All values include D4 dispersion corrections. Figure S1: Correlation between errors relative to the reference values for all reactions in GMTKN55. Table S4: Performance of selected rung 4 functionals in self‐consistent or post‐SCF calculations for the mean absolute deviation (MAD) of the W4‐11RE reaction‐energy test set. Figure S2: Comparison of nP‐LMF plots obtained with the EC(LH) protocol using PBE orbitals and from fully self‐consistent LH25nP calculations. Figure S3: Spin‐restricted potential energy curves for selected diatomic molecules. Figure S4: Errors in the NaCl stretching energy, ΔEerror[NaCl(2.4˚A) − NaCl(6.4˚A)], in kcal mol−1, evaluated with respect to CCSD(T) reference data. Table S5: Wall times (s), number of SCF cycles, and speed‐up of EC(LH25nP)@DFA compared to self‐consistent LH25nP for energy calculations on a series of oligoacene systems with increasing number of rings (in D 2h symmetry) and for C60 in C 1 symmetry. [file JCC-47-0-s001.pdf]

An energy-corrected fast post-SCF local-hybrid scheme for highly accurate energy differences of large main-group systems

Artur Wodyński,* Martin Kaupp*

Correspondence.
Artur Wodyński, Technische Universität Berlin, Institute of Chemistry, Theoretical Chemistry/Quantum Chemistry, Sekr. C7, Straße des 17. Juni 135, 10623 Berlin, Germany. Email: artur.wodynski@tu-berlin.de.
Martin Kaupp, Technische Universität Berlin, Institute of Chemistry, Theoretical Chemistry/Quantum Chemistry, Sekr. C7, Straße des 17. Juni 135, 10623 Berlin, Germany. Email: martin.kaupp@tu-berlin.de.

Table S1. Mean absolute deviations (kcal/mol) for GMTKN55 subsets self-consistent LH25nP (LH25nP@LH25nP; gridsize m4), LH25nP (gridsize 4) on SVWN orbitals (LH25nP@SVWN), and the energy-corrected LH25nP variant on PBE orbitals (EC(LH25nP)@SVWN), using gridsize 2, 3, and 4. All values include D4 dispersion corrections.

	LH25nP @SVWN	LH25nP[S1] @LH25nP	EC(LH25nP)-g2 @SVWN	EC(LH25nP)-g3 @SVWN	EC(LH25nP)-g4 @SVWN
ACONF	0.14	0.03	0.08	0.05	0.03
ADIM6	0.66	0.05	0.29	0.29	0.37
AHB21	1.04	0.78	0.55	0.58	0.54
AL2X6	3.92	1.10	1.60	2.67	1.39
ALK8	5.95	4.84	3.71	3.51	3.88
ALKBDE10	5.75	3.84	4.41	4.97	4.99
AMINO20x4	0.16	0.14	0.16	0.13	0.11
BH76	1.40	0.79	0.84	0.79	0.73
BH76RC	1.35	0.82	0.92	0.77	0.75
BHDIV10	1.33	1.04	1.24	1.52	1.32
BHPERI	0.97	0.64	0.77	0.79	0.67
BHROT27	0.23	0.27	0.28	0.23	0.24
BSR36	0.72	0.44	0.46	0.45	0.40
BUT14DIOL	0.19	0.08	0.13	0.07	0.05
C60ISO	12.24	15.01	16.48	7.97	12.30
CARBHB12	0.82	0.24	0.45	0.49	0.51
CDIE20	0.21	0.25	0.23	0.20	0.17
CHB6	1.20	1.09	0.99	0.79	0.88
DARC	0.93	0.47	0.40	0.58	0.41
DC13	4.19	2.66	4.73	4.21	3.40
DIPCS10	12.36	11.70	12.92	13.11	13.05
FH51	0.69	0.89	1.00	1.02	1.00
G21EA	2.95	2.61	3.27	3.40	3.32
G21IP	5.72	5.42	5.74	5.70	5.49
G2RC	1.95	1.60	1.92	1.63	1.55
HAL59	0.52	0.27	0.23	0.19	0.24
HEAVY28	0.12	0.10	0.14	0.14	0.12
HEAVYSB11	2.62	1.88	2.30	2.37	2.18
ICONF	0.18	0.16	0.18	0.13	0.22
IDISP	1.79	0.86	1.39	1.16	1.02
IL16	2.07	1.24	0.98	0.75	0.92
INV24	1.15	0.95	0.92	0.78	0.94
ISO34	0.59	0.43	0.32	0.38	0.40
ISOL24	1.67	1.31	1.54	1.54	1.58
MB16-43	25.90	20.24	16.20	17.71	17.76
MCONF	0.10	0.13	0.15	0.13	0.13
NBPRC	2.16	1.11	0.94	0.86	1.02
PA26	3.68	3.67	4.22	4.38	4.27
PArel	0.63	0.50	0.51	0.57	0.54

	LH25nP @SVWN	LH25nP @LH25nP	EC(LH25nP)-g2 @SVWN	EC(LH25nP)-g3 @SVWN	EC(LH25nP)-g4 @SVWN
PCONF21	0.17	0.36	0.38	0.32	0.26
PNICO23	0.75	0.17	0.20	0.12	0.15
PX13	4.35	3.97	2.47	3.80	3.15
RC21	1.77	1.56	1.63	1.42	1.49
RG18	0.24	0.04	0.06	0.09	0.08
RSE43	0.86	0.36	0.55	0.52	0.50
S22	0.44	0.19	0.15	0.12	0.11
S66	0.41	0.13	0.21	0.18	0.18
SCONF	0.14	0.14	0.27	0.20	0.24
SIE4x4	6.21	5.85	7.01	6.64	7.00
TAUT15	0.54	0.56	0.40	0.38	0.40
UPU23	0.51	0.41	0.50	0.40	0.35
W411	2.97	2.10	1.57	1.49	1.48
WATER27	4.86	1.35	1.37	1.42	1.39
WCPT18	1.19	1.26	1.45	1.84	1.44
YBDE18	1.81	1.50	1.38	1.42	1.50

Table S2. Mean absolute deviations (kcal/mol) for GMTKN55 subsets self-consistent LH25nP (LH25nP@LH25nP; gridsize m4), LH25nP (gridsize 4) on PBE orbitals (LH25nP@PBE), and the energy-corrected LH25nP variant on PBE orbitals (EC(LH25nP)@PBE), using gridsize 2, 3, and 4. All values include D4 dispersion corrections.

	LH25nP @PBE	LH25nP[S1] @LH25nP	EC(LH25nP)-g2 @PBE	EC(LH25nP)-g3 @PBE	EC(LH25nP)-g4 @PBE
ACONF	0.05	0.03	0.08	0.05	0.04
ADIM6	0.25	0.05	0.04	0.10	0.13
AHB21	0.93	0.78	0.61	0.60	0.63
AL2X6	2.80	1.10	1.48	2.22	1.20
ALK8	5.16	4.84	3.61	3.33	4.06
ALKBDE10	5.35	3.84	4.36	4.87	4.72
AMINO20x4	0.18	0.14	0.16	0.15	0.11
BH76	1.20	0.79	0.79	0.75	0.73
BH76RC	1.17	0.82	0.84	0.76	0.67
BHDIV10	1.16	1.04	1.28	1.40	1.36
BHPERI	0.90	0.64	0.56	0.60	0.59
BHROT27	0.23	0.27	0.27	0.25	0.27
BSR36	0.48	0.44	0.40	0.48	0.37
BUT14DIOL	0.16	0.08	0.14	0.06	0.07
C60ISO	11.94	15.01	17.10	8.28	12.47
CARBHB12	0.54	0.24	0.33	0.36	0.42
CDIE20	0.23	0.25	0.25	0.21	0.19
CHB6	1.09	1.09	1.02	0.77	0.87
DARC	0.30	0.47	0.37	0.65	0.33
DC13	3.68	2.66	5.08	4.18	3.15
DIPCS10	11.91	11.70	12.30	12.02	12.44
FH51	0.80	0.89	1.08	0.92	0.99
G21EA	2.91	2.61	3.23	3.18	3.18
G21IP	5.49	5.42	5.30	5.36	5.23
G2RC	2.07	1.60	1.88	1.57	1.58
HAL59	0.37	0.27	0.23	0.19	0.21
HEAVY28	0.10	0.10	0.11	0.11	0.09
HEAVYSB11	2.41	1.88	2.53	2.40	2.28
ICONF	0.17	0.16	0.15	0.10	0.17
IDISP	1.16	0.86	1.44	0.88	0.85
IL16	1.79	1.24	1.19	0.94	1.04
INV24	1.41	0.95	1.35	1.32	1.30
ISO34	0.51	0.43	0.32	0.39	0.41
ISOL24	1.32	1.31	1.52	1.43	1.52
MB16-43	24.28	20.24	14.27	17.21	15.75
MCONF	0.11	0.13	0.13	0.12	0.12
NBPRC	1.64	1.11	1.09	1.07	1.14
PA26	3.70	3.67	4.46	4.39	4.41
PArel	0.51	0.50	0.43	0.59	0.50

	LH25nP @PBE	LH25nP @LH25nP	EC(LH25nP)-g2 @PBE	EC(LH25nP)-g3 @PBE	EC(LH25nP)-g4 @PBE
PCONF21	0.21	0.36	0.39	0.35	0.29
PNICO23	0.50	0.17	0.15	0.12	0.13
PX13	4.12	3.97	1.47	3.49	3.15
RC21	1.49	1.56	1.59	1.29	1.55
RG18	0.08	0.04	0.06	0.08	0.06
RSE43	0.61	0.36	0.54	0.50	0.47
S22	0.31	0.19	0.16	0.17	0.15
S66	0.24	0.13	0.19	0.17	0.16
SCONF	0.19	0.14	0.31	0.24	0.22
SIE4x4	6.34	5.85	6.63	6.00	6.54
TAUT15	0.39	0.56	0.41	0.44	0.45
UPU23	0.45	0.41	0.48	0.46	0.39
W411	2.62	2.10	1.68	1.44	1.49
WATER27	3.50	1.35	2.84	2.43	1.83
WCPT18	1.01	1.26	1.22	1.58	1.41
YBDE18	1.68	1.50	1.54	1.52	1.49

Table S3. Mean absolute deviations (kcal/mol) for GMTKN55 subsets self-consistent LH25nP (LH25nP@LH25nP; gridsize m4), LH25nP (gridsize 4) on r^2SCAN orbitals (LH25nP@r^2SCAN), and the energy-corrected LH25nP variant on PBE orbitals (EC(LH25nP)@r^2SCAN), using gridsizse 3, and 4. All values include D4 dispersion corrections.

	LH25nP @r^2SCAN	LH25nP^{S1} @LH25nP	EC(LH25nP)-g2 @r^2SCAN	EC(LH25nP)-g3 @r^2SCAN	EC(LH25nP)-g4 @r^2SCAN
ACONF	0.03	0.03	0.07	0.04	0.04
ADIM6	0.09	0.05	0.05	0.09	0.09
AHB21	0.77	0.78	0.61	0.53	0.65
AL2X6	1.91	1.10	1.36	2.23	1.20
ALK8	4.65	4.84	3.74	3.10	3.76
ALKBDE10	4.57	3.84	4.36	4.83	4.80
AMINO20x4	0.15	0.14	0.15	0.15	0.13
BH76	1.08	0.79	0.83	0.77	0.79
BH76RC	1.15	0.82	0.71	0.73	0.69
BHDIV10	1.08	1.04	1.21	1.26	1.23
BHPERI	0.82	0.64	0.58	0.53	0.57
BHROT27	0.22	0.27	0.25	0.25	0.28
BSR36	0.39	0.44	0.42	0.60	0.57
BUT14DIOL	0.11	0.08	0.12	0.06	0.06
C60ISO	10.86	15.01	15.72	7.25	11.37
CARBHB12	0.30	0.24	0.21	0.23	0.26
CDIE20	0.23	0.25	0.25	0.20	0.19
CHB6	1.05	1.09	1.08	0.88	0.92
DARC	0.36	0.47	0.59	0.49	0.34
DC13	3.16	2.66	4.72	4.04	3.00
DIPCS10	11.83	11.70	12.61	12.31	12.86
FH51	0.87	0.89	0.95	0.86	0.85
G21EA	2.76	2.61	3.13	3.12	3.22
G21IP	5.39	5.42	5.29	5.39	5.30
G2RC	1.92	1.60	1.66	1.33	1.49
HAL59	0.28	0.27	0.20	0.18	0.19
HEAVY28	0.08	0.10	0.11	0.09	0.08
HEAVYSB11	1.88	1.88	2.14	2.06	2.24
ICONF	0.15	0.16	0.13	0.10	0.15
IDISP	0.74	0.86	1.30	0.90	0.81
IL16	1.50	1.24	1.04	0.89	0.97
INV24	1.23	0.95	1.25	1.20	1.24
ISO34	0.47	0.43	0.35	0.39	0.45
ISOL24	1.15	1.31	1.63	1.45	1.56
MB16-43	16.86	20.24	10.96	11.55	11.36
MCONF	0.13	0.13	0.14	0.13	0.13
NBPRC	1.35	1.11	0.96	0.89	0.99
PA26	3.65	3.67	4.42	4.46	4.49
PArel	0.55	0.50	0.49	0.51	0.54

	LH25nP @r^{2}SCAN	LH25nP @LH25nP	EC(LH25nP)-g2 @r^{2}SCAN	EC(LH25nP)-g3 @r^{2}SCAN	EC(LH25nP)-g4 @r^{2}SCAN
PCONF21	0.37	0.36	0.43	0.40	0.33
PNICO23	0.32	0.17	0.12	0.10	0.10
PX13	4.01	3.97	1.51	3.46	2.46
RC21	1.63	1.56	1.77	1.51	1.78
RG18	0.10	0.04	0.08	0.08	0.08
RSE43	0.51	0.36	0.51	0.48	0.45
S22	0.22	0.19	0.15	0.13	0.13
S66	0.13	0.13	0.16	0.13	0.13
SCONF	0.13	0.14	0.25	0.23	0.18
SIE4x4	6.11	5.85	6.49	5.99	6.47
TAUT15	0.40	0.56	0.52	0.41	0.44
UPU23	0.42	0.41	0.45	0.41	0.37
W411	2.26	2.10	1.57	1.46	1.49
WATER27	2.40	1.35	2.34	2.17	1.35
WCPT18	1.02	1.26	1.08	1.46	1.20
YBDE18	1.63	1.50	1.54	1.55	1.62

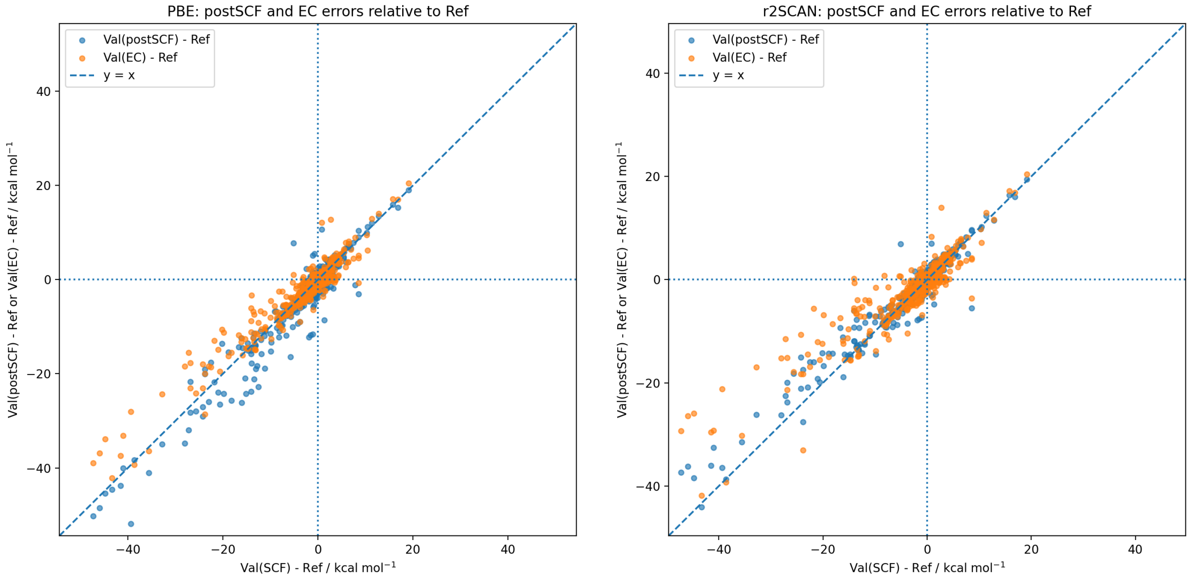

Figure S1. Correlation between errors relative to the reference values for all reactions in GMTKN55. The horizontal axis shows the self-consistent error, Val(SCF)-Ref, while the vertical axis shows the corresponding post-SCF and energy-corrected EC errors obtained using PBE and r^{2}SCAN orbitals, Val(postSCF)-Ref and Val(EC)-Ref, respectively, all in kcal mol^{-1}. The dashed diagonal line indicates equal errors for the non-self-onsistent and self-consistent calculations; therefore, points closer to this line have smaller deviations from the SCF results. In contrast, points closer to the horizontal zero-error axis have smaller errors with respect to the reference data. The dotted horizontal and vertical lines mark zero error.

Table S4. Performance of selected rung 4 functionals in self-consistent or post-SCF clculations for the mean absolute deviation (MAD) of the W4-11RE reaction-energy test set.

	MAD [kcal/mol]
M05-2X	4.7[c]
ωB97M-V	3.3[c]
LH25nP-D4	2.4[b]
LH25nP-D4@SVWN	2.9[a]
EC(LH25nP-D4)@SVWN	2.2[a]
LH25nP-D4@PBE	2.7[a]
EC(LH25nP-D4)@PBE	2.2[a]
LH25nP-D4@r^{2}SCAN	2.7[a]
EC(LH25nP-D4)@r^{2}SCAN	2.2[a]

[a]This work. [b] Ref. S1. [c]Ref. S3.

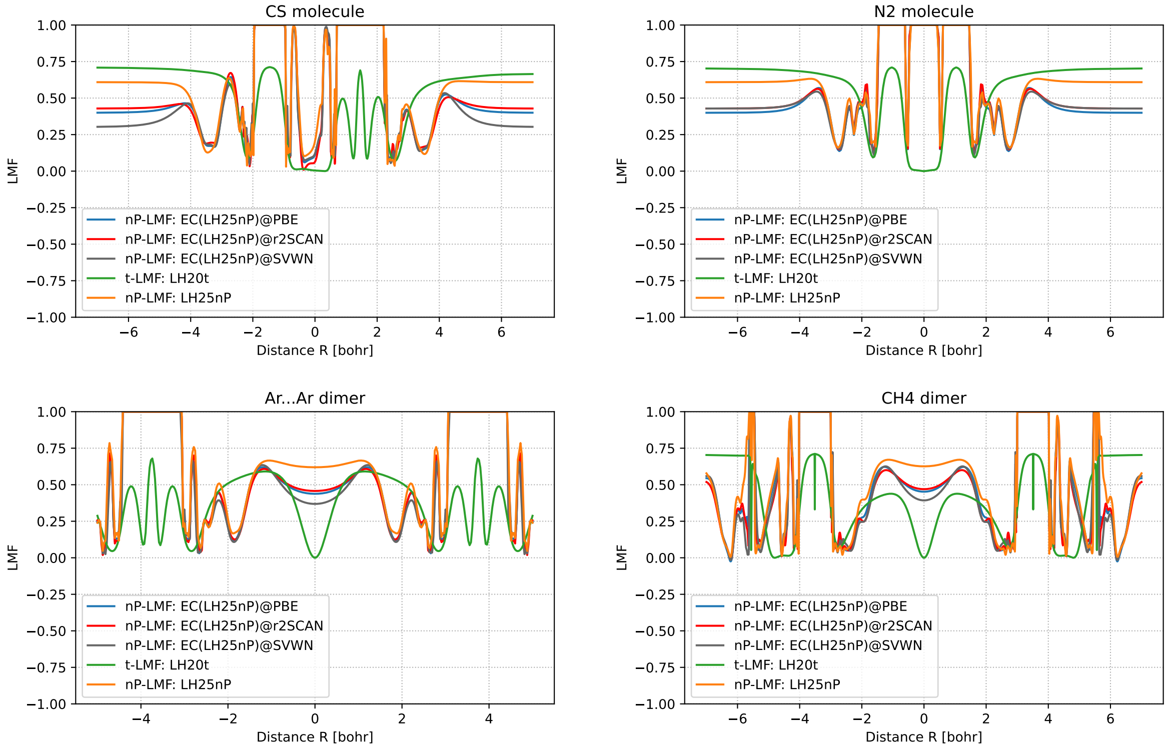

Figure S2. Comparison of nP-LMF plots obtained with the EC(LH) protocol using PBE orbitals and from fully self-consistent LH25nP calculations. The t-LMF of LH20t is included for comparison. Results are shown for CS, N_2, the Ar dimer at an internuclear distance of 7.5 bohr, and $(CH_4)_2$ at a C–C internuclear distance of 7.0 bohr, i.e., systems and geometries for which gauge-related problems are usually most severe.

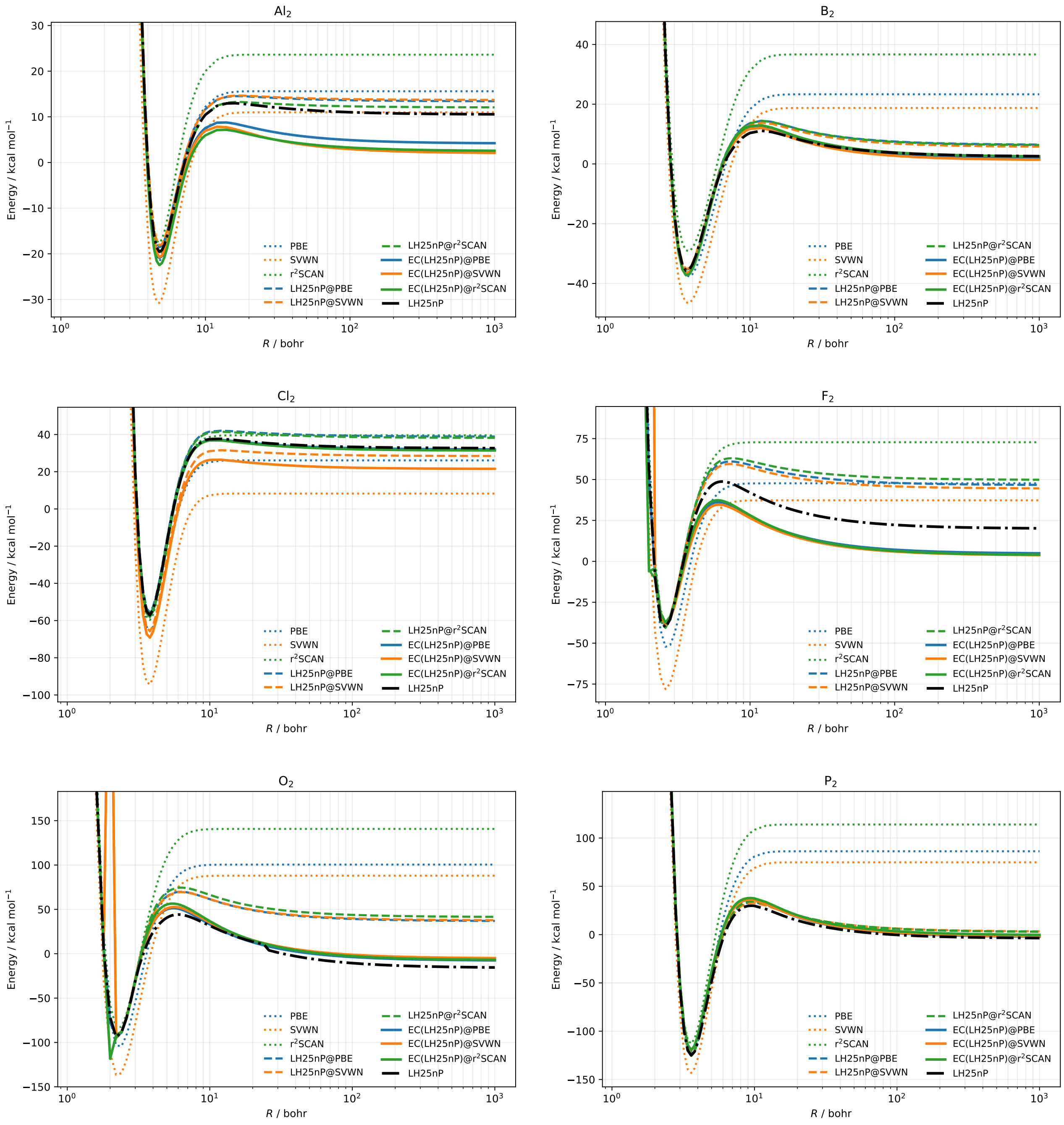

Figure S3. Spin-restricted potential energy curves for selected diatomic molecules.

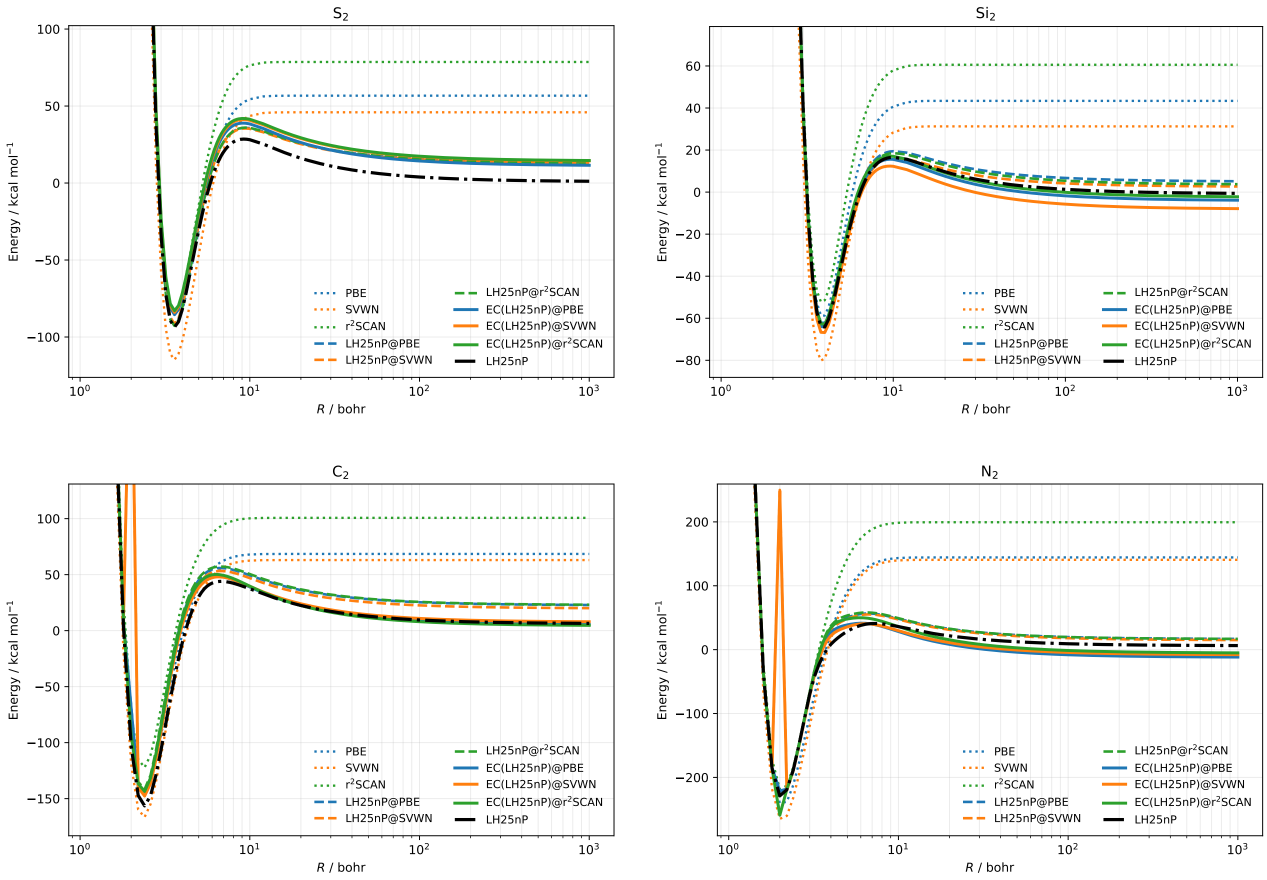

Figure S3. Spin-restricted potential energy curves for selected diatomic molecules, continued.

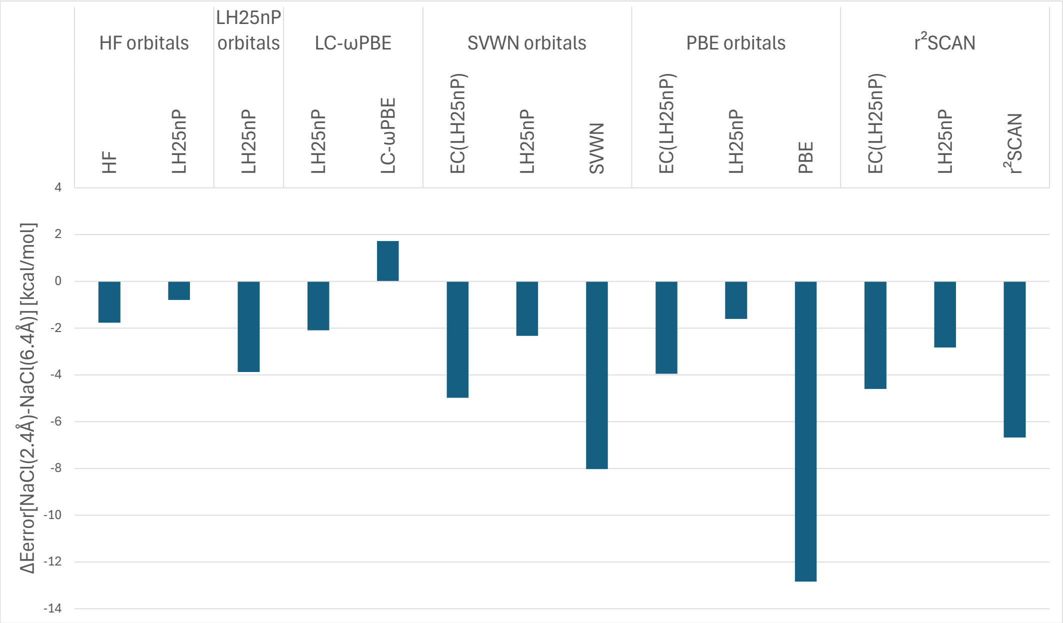

Figure S4. Errors in the NaCl stretching energy, $\Delta E_{\text{error}}[\text{NaCl}(2.4\,\text{Å}) - \text{NaCl}(6.4\,\text{Å})]$, in kcal mol^{-1}, evaluated with respect to CCSD(T) reference data.[S2] Results are shown for HF, self-consistent LH25nP, and for LH25nP and retrained EC(LH25nP) evaluated non-self-consistently on different sets of orbitals/densities. The non-self-consistent calculations are grouped according to the orbitals used in the energy evaluation: HF, LH25nP, LC-ωPBE, SVWN, PBE, and r^2SCAN orbitals. For the semilocal reference densities, both the original non-retrained LH25nP@DFA and the retrained EC(LH25nP)@DFA variants are shown, where DFA = SVWN, PBE, or r^2SCAN. This quantity is assumed to reflect large density-driven errors at the stretched structure. All calculations use the same DFT-D4 parameters.

Table S5. Wall times (s), number of SCF cycles, and speed-up of EC(LH25nP)@DFA compared to self-consistent LH25nP for energy calculations on a series of oligoacene systems with increasing number of rings (in D_{2h} symmetry) and for C_{60} in C_1 symmetry.[a] All timings are for 10 cores of Intel Xeon E5-2630 v4 CPU.

	SCF (LH25nP)		SCF(PBE)		SCF(r^{2}SCAN)		EC(LH25nP) [s]			speed-up[c]		
acene[b]	[s]	cycles	[s]	cycles	[s]	cycles	g2	g3	g4	g2	g3	g4
3	128	13	7	12	10	12	8	13	25	8.5	6.3	4.0
4	198	12	12	11	15	12	14	23	43	7.8	5.7	3.6
5	319	13	21	13	22	12	21	34	64	7.8	5.8	3.8
6	443	13	25	13	31	13	28	47	88	8.4	6.2	3.9
7	853	20	56	22	69	24	37	63	117	9.2	7.2	4.9
8	1094	20	57	18	70	19	48	79	150	10.4	8.0	5.3
9	1358	19	72	18	91	20	59	99	187	10.4	7.9	5.3
10	1641	20	92	19	103	20	74	124	232	9.9	7.6	5.1
11	2114	21	118	20	131	20	88	148	278	10.3	8.0	5.3
12	2902	25	136	20	-[d]	-[d]	106	173	328	12.0	9.4	6.3
C_{60}	30073	22	1145	20	1106	16	1159	1936	3501	13.1	9.8	6.5

[a]PBE, r^{2}SCAN, and LH25nP SCF with gridsize m4, def2-QZVP basis sets. [b]Number of rings. [c]Speedup calculated as the ratio between wall times of SCF(PBE)+EC(LH25nP) cost vs. full LH25nP SCF wall time. [d]r^{2}SCAN calculations did not converge with the same settings used for the PBE calculations.

References

[S1] A. Wodyński and M. Kaupp, J. Comput. Chem. **47**, e70294 (2026).

[S2] S. Nam, S. Song, E. Sim, and K. Burke, J. Chem. Theory Comput. **16**, 5014 (2020).

[S3] A. Wodyński and M. Kaupp, J. Chem. Theory Comput. **21**, 7419 (2025).
